# Tree diversity of the Dja Faunal Reserve, southeastern Cameroon

**DOI:** 10.3897/BDJ.2.e1049

**Published:** 2014-01-24

**Authors:** Bonaventure Sonké, Thomas L.P. Couvreur

**Affiliations:** † Université de Yaoundé I, Ecole Normale Supérieure, Yaoundé, Cameroon; ‡ Institut de Recherche pour le Développement (IRD), Montpellier, France

**Keywords:** Africa, diversity, transect, Dja Faunal Reserve, conservation, species inventory

## Abstract

The Dja Faunal Reserve located in southeastern Cameroon represents the largest and best protected rainforest patch in Cameroon. Here we make available a dataset on the inventory of tree species collected across the Dja. For this study nine 5 km long and 5 m wide transects were installed. All species with a diameter at breast height greater than 10 cm were recorded, identified and measured. A total of 11546 individuals were recorded, corresponding to a total of 312 species identified with 60 genera containing unidentified taxa. Of the 54 identified families Fabaceae, Rubiaceae and Malvaceae were the most species rich, whereas Fabaceae, Phyllantaceae and Olacaceae were the most abundant. Finally, *Tabernaemontana crassa* was the most abundant species across the Reserve. This dataset provides a unique insight into the tree diversity of the Dja Faunal Reserve and is now publically available and usable.

## Introduction

Tropical rainforests are among the most diverse plant communities worldwide and contain more than 50% of the Earth’s species diversity (Parmentier et al. 2007). Rain forests are remarkable by the diversity of tree species they contain, in places reaching up to 300 different species within a single hectare of inventoried forest (Valencia et al. 1994). Yet inventory of species in tropical countries remains challenging (Campbell and Hammond 1989). The Republic of Cameroon, situated on the Atlantic Coast in the Gulf of Guinea, covers 475440 km² and has such a great diversity of habitats that it is often compared to "a small Africa".

Although Cameroon has been better explored by botanists than most of other Central African countries (Letouzey 1968), its flora remains incompletely known, and several new species are described or newly recorded every year (e.g. Cheek 2009, Droissart et al. 2009, Lachenaud and Séné 2012, Sonké et al. 2009, Lachenaud et al. 2013). This is partly due to the high botanical diversity of the country (e.g. Kenfack et al. 2007) which includes many endemics (Onana 2011). In addition, botanical efforts have tended to focus on some specific sites (e.g. Mt Cameroon, Bakossi mountains, Campo Ma'an National Park; Cheek et al. 2004, Cable and Cheek 1998, Tchouto et al. 2006), leaving other areas almost unexplored, especially in the west and southeast (Onana 2011, Droissart et al. 2012). The present contribution publishes the results of an inventory made some years ago in the Dja Faunal Reserve, a UNESCO World Heritage Site, located in southeastern Cameroon. Its altitude is between 500 and 700 m and covers an area of 526,000 ha mainly classified as moist evergreen tropical forests  (Letouzey 1985) and belongs to the Guineo-congolian phytochoria of (White 1979). It is bounded on three-quarters of its periphery by a natural barrier that is the Dja river. The reserve also belongs to the TRIDOM forest block that encompasses several other major reserves such as Odzala National Parc in the République du Congo and the Minkébé Natinal Parc in Gabon. This region is characterized by a low collection density of botanical specimens and are thus fragmentarily known (Valkenburg et al. 1998, Onana 2011, Droissart et al. 2012).

The present dataset was available only under printed form (Sonké 2004, Sonké 1998). Here we make it available open access under electronic form. This dataset has been used to assess the role of dispersal and niche differentiation in explaining species distribution across the reserve (Hardy and Sonké 2004).

## Project description

### Title

Tree Inventory of the Dja Faunal Reserve, South Cameroon.

### Personnel

Bonaventure Sonké

### Study area description

**Figure 1.**
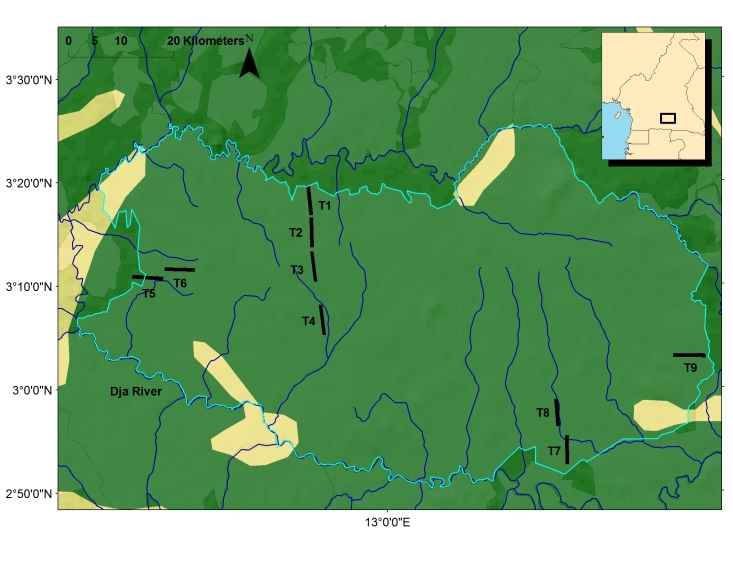
The Dja Faunal Reserve, South Cameroon, with the location of the 9 transects (T1-T9). Dark blue lines represent rivers. Light blue lines represent the delimitation of the Dja Faunal Reserve. Yellow patches are degraded vegetation while green and dark green patches represent tropical rain forest. Finer scale vegetation patterns are note represented here. Vegetation types based on Guillaumet, Chevillotte & Valton (IRD; MNHN).

The dataset contains tree inventory of nine 5 km long 5 meters wide transects across the Dja Faunal Reserve. The Dja Forest Reserve is situated at 2°50 – 3°30 N and 12°20 – 13°40 E in southeastern Cameroon, bounded on three sides by the Dja River (Fig. 1), a major tributary of the Congo river, within the area known as the southern plateau. Most of the Dja Faunal Reserve is situated at 600–700 m above the sea level and covers 526,000 ha making it one of the largest and best protected areas of lowland rainforest across tropical Africa. The monthly average temperature lies between 23.5 °C and 24.5 °C and the annual rainfall between 1180 mm and 2350 mm. There are four major vegetation types in the Dja: primary *terra firme* primary forest (74% of total individuals collected occurring in this vegetation type); (ii) secondary forest (8% of total individuals); (iii) gaps (4% of total individuals). Finally, two hydromorphic types were defined as (iv) swamps (11%) and (v) flooded forest (3%). The different vegetation types across each transect are provided in Suppl. material 2. Transects were placed at the following coordinates:

T1: start: 12.8573; 3.3243; end: 12.8622; 3.2824

T2: start: 12.8637; 3.2303; end: 12.8622; 3.2726

T3: start: 12.8695; 3.1752; end: 12.8636; 3.2183

T4: start: 12.8846; 3.0895; end: 12.8787; 3.1325

T5: start: 12.5367; 3.1816; end: 12.5866; 3.1787

T6: start: 12.5958; 3.1936; end: 12.6447; 3.1925

T7: start: 13.3293; 2.9203; end: 13.3301; 2.8792

T8: start: 13.3105; 2.9787; end: 13.3133; 2.9403

T9: start: 13.5287; 3.0510; end: 13.5817; 3.0498

There are local Baka people living in the forest practicing subsistence hunting and gathering, thus there is some minimal human pressure on the forest. The forest has its full compliment of large animals, including Western Lowland Gorillas (*Gorilla gorilla gorilla*) and African forest elephants (*Loxodonto Africana cyclotis*) (Dupain et al. 2004).

### Funding

Collection of the data in the field was funded by the European Union project ECOFAC.

## Sampling methods

### Study extent

The dataset was collected between August 1993 and August 1995.

### Sampling description

A line transect approach was chosen to sample forest diversity because it covers the heterogeneity of the forest better than a square plot. We used standardized methods to install the transects, identify, map, measure and re-measure the trees (Baker et al. 2004). Each transect line was 5 meters wide and 5 km long. A total of 9 such transects were done at different places across the reserve Fig. 1. All trees ≥ 10 cm dbh (diameter at breast height, 1.3 m or above all deformities, notable buttresses, if present), were identified, mapped, individually numbered with an aluminium tag and nail, and measured with a diameter tape, or estimated if this the correct point of measurement was not possible to reach. Trees were identified up to species level, and in some cases just up to genus level. Voucher specimens were collected and deposited in different herbaria (mainly YA) and in case of any doubt, supplementary material was collected for further identifications.

### Quality control

The present dataset was updated to match the APG III classification of angiosperm families (APGIII 2003) and all species names were checked for validity (spelling and authorship) against online databses (http://tnrs.iplantcollaborative.org/index.html, http://ipni.org; http://plants.jstor.org/; http://www.ville-ge.ch/musinfo/bd/cjb/africa/recherche.php).

### Step description

The dataset presented here was collected over a period of three years.

## Geographic coverage

### Description

Data was collected in 9 different sites across the reserve as illustrated in Fig. 1.

### Coordinates

13.666667 and 2.833333 Latitude; 12.333333 and 3.5 Longitude.

## Taxonomic coverage

### Description

The dataset contains a total of 11546 tagged individuals representing 312 total taxa identified to species level (Table 1) belonging to 54 families and 213 genera (a few photos of selected species are provided in Figs 2, 3). For 60 genera some individuals remained unidentified (marked as sp.). This is mainly related to the poorly known taxonomy of those genera or lack of appropriate material (e.g. flowers) to provide a definite determination. The raw data is available in Suppl. material 1.

**Figure 4.**
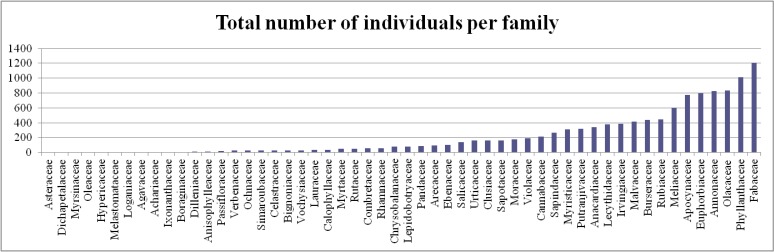
Plant family abundance in the Dja Faunal Reserve. This table was produced from Suppl. material 3.

Of the 54 families Fabaceae, Rubiaceae and Malvaceae were the most species rich (Table 2) whereas Fabaceae, Phyllantaceae and Ochnaceae were the most abundant families in terms of individuals sampled (Table 3, Fig. 4, Suppl. material 3). Finally, *Tabernaemontana crassa* (Apocynaceae) was the most abundant species recorded for the reserve with 633 individuals tagged (Table 4). Species diversity and abundance per family for each transect is given in Table 5.

## Traits coverage

The only trait measured and published in this dataset is the diameter at breast height for each individual found in Suppl. material 1.

## Temporal coverage

**Data range:** 1993 8 01 – 1995 8 31.

## Usage rights

### Use license

Creative Commons CCZero

### IP rights notes

This dataset can be freely used provided it is cited

## Data resources

### Data package title

Djatrees: Trees of the Dja Faunal Reserve

### Resource link

https://sites.google.com/site/tlpcouvreur/home/ressources

### Number of data sets

1

### Data set 1.

#### Data set name

Djatrees

#### Data format

excel sheet

#### Number of columns

4

#### Description

List of all individuals (including sp.) per transect collected in the nine transects.

**Data set 1.** 

Column label	Column description
Species_name	Name of the identified species
Family_name	Plant family for the species follwing APG III
Transect_number	Transect number
Diameter	Diameter at breast hight (in cm)

## Supplementary Material

Supplementary material 1 List of all individuals collected in all 9 transects of the Dja Faunal Resreve. Data type: List of individuals collected in each transcet in the Dja Faunal Reserve
Brief description: This is the raw dataset with indication of each individual collected as well as its diameter. This dataset is also availible at https://sites.google.com/site/tlpcouvreur/home/ressources.
File: oo_5683.xls Sonké & Couvreur

Supplementary material 2 Vegetation distribution across the transects in the Dja Faunal Reserve Cameroon Data type: excel sheet
File: oo_5684.xls Sonké & Couvreur

Supplementary material 3 Individual abundances per family Data type: excel sheet
Brief description: This sheet provides the number of individuals per family for all the species collected in the Dja Faunal Reserve. First coloum: Family; second coloum: number of individuals. this table was used to generate figure 2 of the article.
File: oo_5682.xls Sonké & Couvreur

## Figures and Tables

**Figure 2a.**
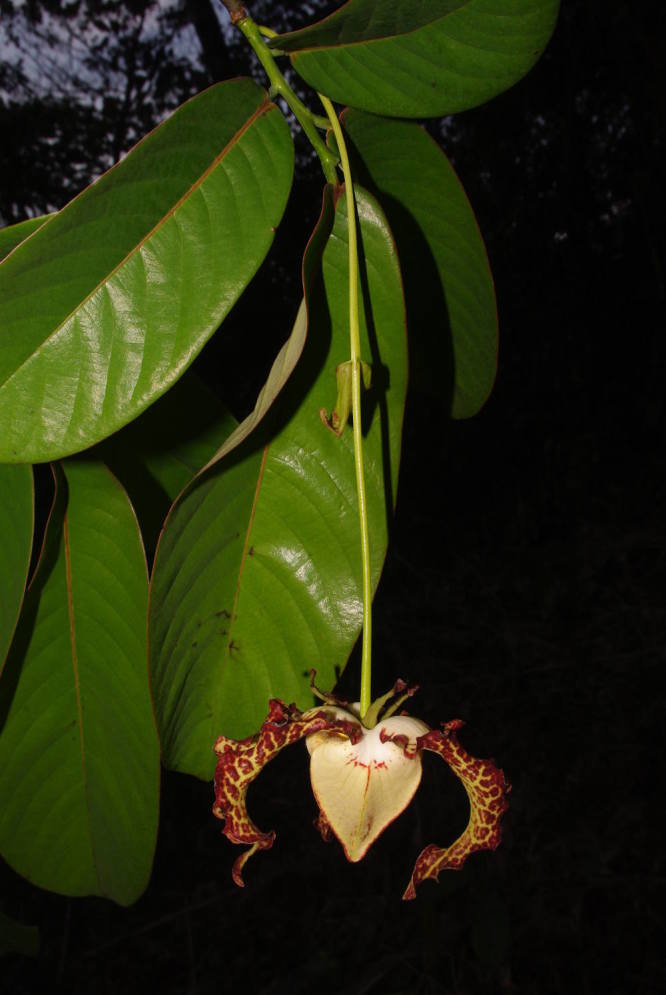
*Monodora myristica* (Annonaceae)

**Figure 2b.**
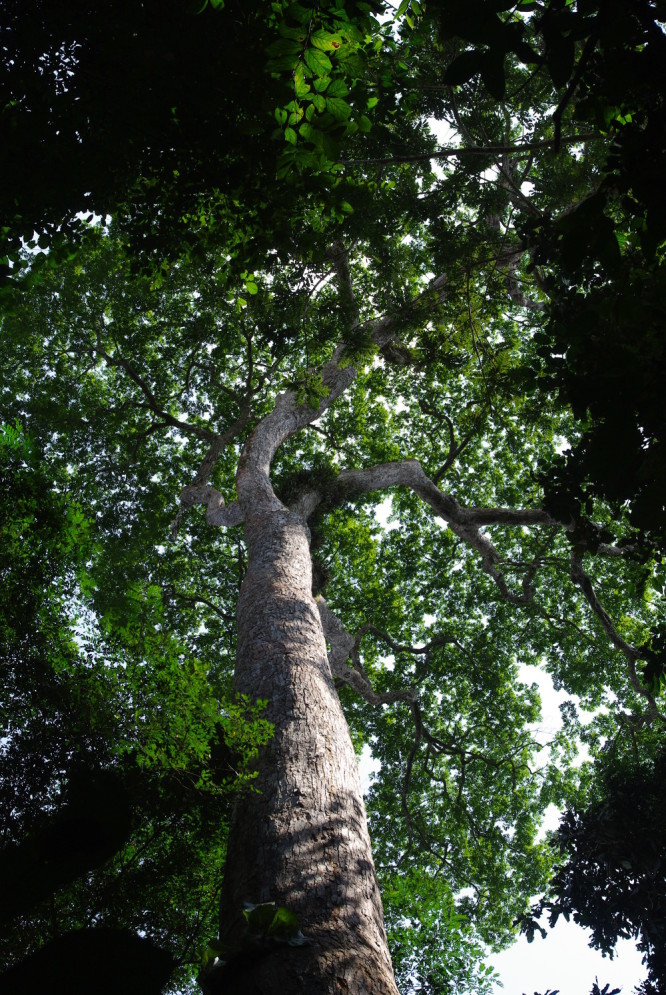
*Amphimas pterocarpoides* (Fabaceae)

**Figure 3a.**
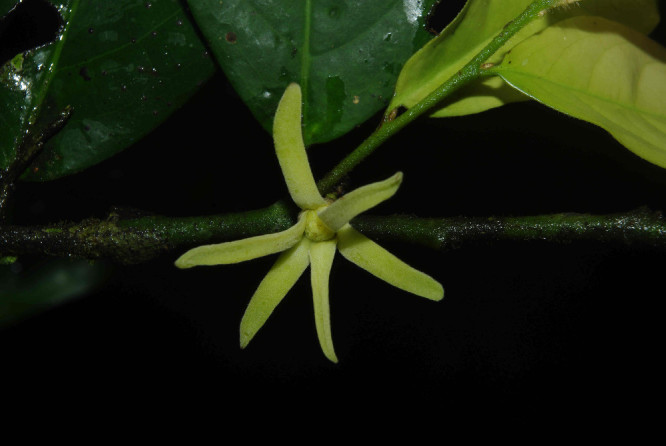
*Greenwayodendron suaveolens* (Annonaceae)

**Figure 3b.**
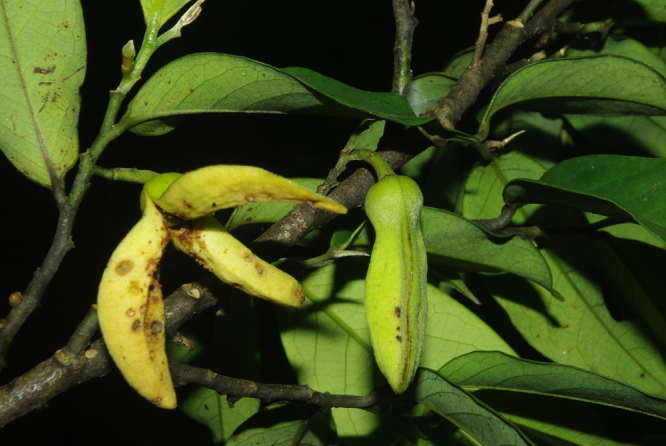
*Annickia affinis* (Annonaceae)

**Figure 3c.**
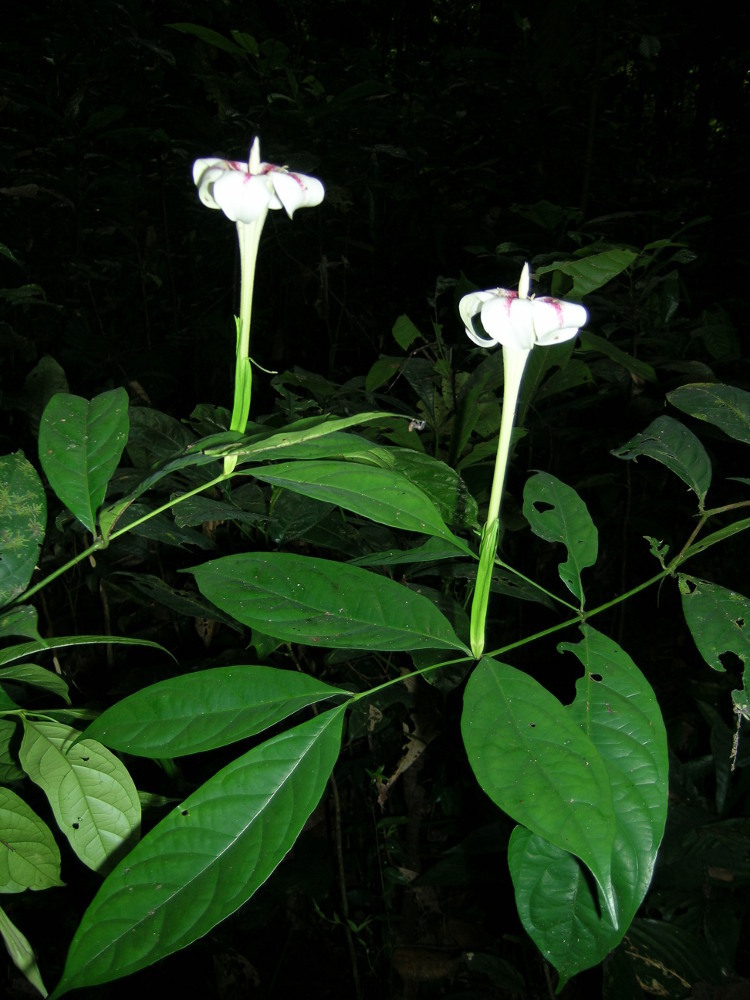
*Rothmannia lateriflora* (Rubiaceae)

**Figure 3d.**
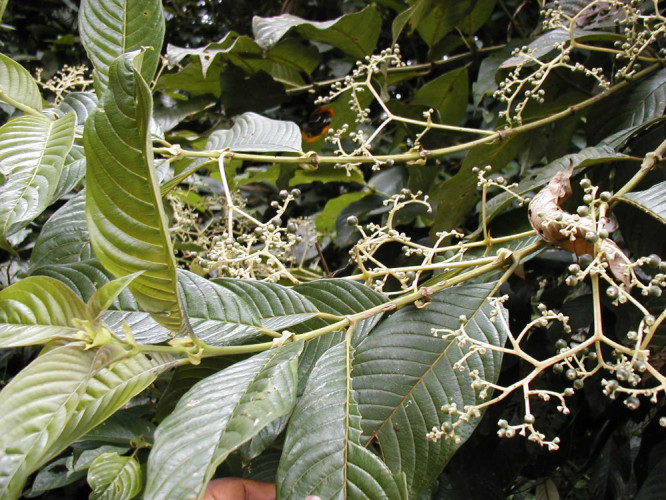
*Pauridiantha floribunda* (Rubiaceae)

**Table 1.** List of identified species collected in the Dja Faunal Reserve.

Species_name	Author_name	APGIII_family_name
*Afzelia bella*	Harms	Fabaceae
*Afzelia bipindensis*	Harms	Fabaceae
*Aidia micrantha*	(K. Schum.) Bullock ex F. White	Rubiaceae
*Albizia adianthifolia*	(Schumach.) W. Wight	Fabaceae
*Albizia ferruginea*	(Guill. & Perr.) Benth.	Fabaceae
*Albizia glaberrima*	Benth.	Fabaceae
*Albizia gummifera*	(J.F. Gmel.) C.A. Sm.	Fabaceae
*Albizia zygia*	J.F. Macbr.	Fabaceae
*Allanblackia floribunda*	Oliv.	Clusiaceae
*Allanblackia gabonensis*	Bamps	Clusiaceae
*Allophylus africanus*	P. Beauv.	Sapindaceae
*Alstonia boonei*	De Wild.	Apocynaceae
*Amphimas ferrugineus*	Pierre ex Harms	Fabaceae
*Amphimas pterocarpoides*	Harms	Fabaceae
*Angylocalyx pynaertii*	De Wild.	Fabaceae
*Annickia affinis*	(Exell) Versteegh & Sosef	Annonaceae
*Anonidium mannii*	Engl. & Diels	Annonaceae
*Anopyxis klaineana*	(Pierre) Engl.	Rhizophoraceae
*Anthonotha cladantha*	(Harms) J. Léonard	Fabaceae
*Anthonotha fragrans*	(Baker f.) Exell & Hillc.	Fabaceae
*Anthonotha macrophylla*	P. Beauv.	Fabaceae
*Antidesma laciniatum*	Aubrév.	Phyllanthaceae
*Antidesma membranaceum*	Müll. Arg.	Phyllanthaceae
*Antidesma venosum*	E. Mey. ex Tul.	Phyllanthaceae
*Antrocaryon klaineanum*	Pierre	Anacardiaceae
*Antrocaryon micraster*	A. Chev. & Guillaumin	Anacardiaceae
*Aoranthe cladantha*	(K. Schum.) Somers	Rubiaceae
*Aulacocalyx jasminiflora*	Hook. f.	Rubiaceae
*Autranella congolensis*	(De Wild.) A. Chev.	Sapotaceae
*Baillonella toxisperma*	Pierre	Sapotaceae
*Balanites wilsoniana*	Dawe & Sprague	Zygophyllaceae
Barteria nigritana subsp. fistulosa	(Mast.) Sleumer	Passifloraceae
*Belonophora coriacea*	Hoyle	Rubiaceae
*Bertiera racemosa*	(G. Don) K. Schum.	Rubiaceae
*Blighia sapida*	K.D. Koenig	Sapindaceae
*Blighia welwitschii*	(Hiern) Radlk.	Sapindaceae
*Bombax brevicuspe*	Sprague	Malvaceae
*Bombax buonopozense*	P. Beauv.	Malvaceae
*Brenania brieyi*	(De Wild.) Petit	Rubiaceae
*Bridelia grandis*	Pierre ex Hutch.	Phyllanthaceae
*Bridelia micrantha*	(Hochst.) Baill.	Phyllanthaceae
*Buchnerodendron speciosum*	Gürke	Achariaceae
*Calpocalyx dinklagei*	Harms	Fabaceae
*Canarium schweinfurthii*	Engl.	Burseraceae
*Carapa procera*	DC.	Meliaceae
*Casearia barteri*	Mast.	Salicaceae
*Cavacoa quintasii*	(Pax & K. Hoffm.) Leonard	Euphorbiaceae
*Ceiba pentandra*	(L.) Gaertn.	Malvaceae
*Celtis adolfi-friderici*	Engl.	Cannabaceae
*Celtis gomphophylla*	Baker	Cannabaceae
*Celtis tessmannii*	Rendle	Cannabaceae
*Celtis zenkeri*	Engl.	Cannabaceae
*Centroplacus glaucinus*	Pierre	Centroplacaceae
*Chlamydocola chlamydantha*	(K. Schum.) M. Bodard	Malvaceae
*Chrysophyllum africanum*	A. DC.	Sapotaceae
*Chrysophyllum boukokoense*	(Aubrév. & Pellegr.) L. Gaut.	Sapotaceae
*Chrysophyllum lacourtianum*	De Wild.	Sapotaceae
*Chrysophyllum pruniforme*	Pierre ex Engl.	Sapotaceae
*Chytranthus atroviolaceus*	Baker f. ex Hutch. & Dalziel	Sapindaceae
*Chytranthus macrobotrys*	(Gilg) Exell & Mendonça	Sapindaceae
*Clausena anisata*	(Willd.) Hook. f. ex Benth.	Rutaceae
*Cleistopholis glauca*	Pierre ex Engl. & Diels	Annonaceae
*Cleistopholis patens*	(Benth.) Engl. & Diels	Annonaceae
*Coelocaryon botryoides*	Vermoesen	Myristicaceae
*Coelocaryon preussii*	Warb.	Myristicaceae
*Cola acuminata*	(P. Beauv.) Schott & Endl.	Malvaceae
*Cola altissima*	Engl.	Malvaceae
*Cola ballayi*	Cornu ex Heckel	Malvaceae
*Cola flavo-velutina*	K. Schum.	Malvaceae
*Cola lateritia*	K. Schum.	Malvaceae
*Cola verticillata*	(Thonn.) Stapf ex A. Chev.	Malvaceae
*Cordia aurantiaca*	Baker	Boraginaceae
*Cordia platythyrsa*	Baker	Boraginaceae
*Corynanthe pachyceras*	K. Schum.	Rubiaceae
*Croton oligandrus*	Pierre ex Hutch.	Euphorbiaceae
*Crotonogyne preussii*	Pax	Euphorbiaceae
*Cuviera cuvieroides*	(Wernham) Onana	Rubiaceae
*Cylicodiscus gabunensis*	Harms	Fabaceae
*Dacryodes buettneri*	(Engl.) H.J. Lam	Burseraceae
*Dacryodes edulis*	(G. Don) H.J. Lam	Burseraceae
*Dacryodes klaineana*	(Pierre) H.J. Lam	Burseraceae
*Desbordesia glaucescens*	(Engl.) Tiegh.	Irvingiaceae
*Desplatsia chrysochlamys*	(Mildbr. & Burret) Mildbr. & Burret	Malvaceae
*Desplatsia dewevrei*	(De Wild. & T. Durand) Burret	Malvaceae
*Detarium macrocarpum*	Harms	Fabaceae
*Dialium bipindense*	Harms	Fabaceae
*Dialium dinklagei*	Harms	Fabaceae
*Dialium guineense*	Willd.	Fabaceae
*Dialium zenkeri*	Harms	Fabaceae
*Dichostemma glaucescens*	Pierre	Euphorbiaceae
*Diospyros bipindensis*	Gürke	Ebenaceae
*Diospyros cinnabarina*	(Gürke) F. White	Ebenaceae
*Diospyros conocarpa*	Gürke & K. Schum.	Ebenaceae
*Diospyros crassiflora*	Hiern	Ebenaceae
*Diospyros hoyleana*	F. White	Ebenaceae
*Diospyros suaveolens*	Gürke	Ebenaceae
*Discoglypremna caloneura*	(Pax) Prain	Euphorbiaceae
*Distemonanthus benthamianus*	Baill.	Fabaceae
*Dracaena arborea*	(Willd.) Link	Asparagaceae
*Drypetes aframensis*	Hutch.	Putranjivaceae
*Drypetes africana*	Vahl	Putranjivaceae
*Drypetes afzelii*	(Pax) Hutch.	Putranjivaceae
*Drypetes capillipes*	(Pax) Pax & K. Hoffm.	Putranjivaceae
*Drypetes chevalieri*	Beille	Putranjivaceae
*Drypetes gossweileri*	S. Moore	Putranjivaceae
*Drypetes klainei*	Pierre ex Pax	Putranjivaceae
*Drypetes laciniata*	(Pax) Hutch.	Putranjivaceae
*Drypetes molunduana*	Pax & K. Hoffm.	Putranjivaceae
*Drypetes paxii*	Hutch.	Putranjivaceae
*Drypetes staudtii*	(Pax) Hutch.	Putranjivaceae
*Duboscia macrocarpa*	A. Chev.	Malvaceae
*Duguetia staudtii*	(Engl. & Diels) Chatrou	Annonaceae
*Elaeophorbia grandifolia*	(Haw.) Croizat	Euphorbiaceae
*Entada gigas*	(L.) Fawc. & Rendle	Fabaceae
*Entandrophragma angolense*	(Welw. ex C. DC.) C. DC.	Meliaceae
*Entandrophragma candollei*	Harms	Meliaceae
*Entandrophragma cylindricum*	(Sprague) Sprague	Meliaceae
*Eribroma oblongum*	(Mast.) Pierre ex A. Chev.	Malvaceae
*Eriocoelum macrocarpum*	Gilg ex Radlk.	Sapindaceae
*Erismadelphus exsul*	Mildbr.	Vochysiaceae
*Erythrophleum suaveolens*	(Guill. & Perr.) Brenan	Fabaceae
*Fernandoa adolphi-friderici*	(Gilg & Mildbr.) Heine	Bignoniaceae
*Ficus exasperata*	Vahl	Moraceae
*Ficus mucuso*	Welw. ex Ficalho	Moraceae
*Ficus sur*	Forssk.	Moraceae
*Funtumia africana*	(Benth.) Stapf	Apocynaceae
*Funtumia elastica*	(Preuss) Stapf	Apocynaceae
*Garcinia kola*	Heckel	Clusiaceae
*Garcinia gnetoides*	Hutch. & Dalziel	Clusiaceae
*Garcinia mannii*	Oliv.	Clusiaceae
*Garcinia punctata*	Oliv.	Clusiaceae
*Garcinia smeathmannii*	(Planch. & Triana) Oliv.	Clusiaceae
*Gilbertiodendron dewevrei*	(De Wild.) J. Léonard	Fabaceae
*Glyphaea brevis*	(Spreng.) Monach.	Malvaceae
*Gossweilerodendron joveri*	Normand ex Aubrév.	Fabaceae
*Greenwayodendron suaveolens*	(Engl. & Diels) Verdc.	Annonaceae
*Grewia brunnea*	K. Schum.	Malvaceae
*Grewia coriacea*	Mast.	Malvaceae
*Grewia oligoneura*	Sprague	Malvaceae
*Guarea cedrata*	(A. Chev.) Pellegr.	Meliaceae
*Guarea thompsonii*	Sprague & Hutch.	Meliaceae
*Harungana madagascariensis*	Lam. ex Poir.	Hypericaceae
*Heinsia crinita*	(Afzel.) G. Taylor	Rubiaceae
*Heisteria trillesiana*	Pierre	Olacaceae
*Heisteria zimmereri*	Engl.	Olacaceae
*Hexalobus crispiflorus*	A. Rich.	Annonaceae
*Homalium discophylum*	Jacq.	Salicaceae
*Homalium le-testui*	Pellegr.	Salicaceae
*Hylodendron gabunense*	Taub.	Fabaceae
*Hymenocardia lyrata*	Tul.	Phyllanthaceae
*Hymenostegia afzelii*	(Oliv.) Harms	Fabaceae
*Irvingia excelsa*	Mildbr.	Irvingiaceae
*Irvingia gabonensis*	(Aubry-Lecomte ex O'Rorke) Baill.	Irvingiaceae
*Irvingia grandifolia*	(Engl.) Engl.	Irvingiaceae
*Irvingia robur*	Mildbr.	Irvingiaceae
*Keayodendron bridelioides*	(Gilg & Mildbr. ex Hutch. & Dalziel) Leandri	Phyllanthaceae
*Khaya ivorensis*	A. Chev.	Meliaceae
*Klaineanthus gaboniae*	Pierre ex Prain	Euphorbiaceae
*Klainedoxa gabonensis*	Pierre ex Engl.	Irvingiaceae
*Klainedoxa microphylla*	A.H. Gentry	Irvingiaceae
*Lannea welwitschii*	(Hiern) Engl.	Anacardiaceae
*Lasiodiscus mannii*	Hook. f.	Rhamnaceae
*Lecaniodiscus cupanioides*	Planch. ex Benth.	Sapindaceae
*Lepidobotrys staudtii*	Engl.	Lepidobotryaceae
*Leptactina involucrata*	Hook. f.	Rubiaceae
*Lindackeria echinata*	C. Presl	Achariaceae
*Lovoa trichilioides*	Harms	Meliaceae
*Macaranga barteri*	Müll. Arg.	Euphorbiaceae
*Macaranga occidentalis*	(Müll. Arg.) Müll. Arg.	Euphorbiaceae
*Macaranga spinosa*	Müll. Arg.	Euphorbiaceae
*Maesobotrya klaineana*	(Pierre) J. Léonard	Phyllanthaceae
*Maesopsis eminii*	Engl.	Rhamnaceae
*Mammea africana*	G. Don	Calophyllaceae
*Manilkara letouzei*	Aubrév.	Sapotaceae
*Manilkara obovata*	(Sabine & G. Don) J.H. Hemsl.	Sapotaceae
*Maprounea membranacea*	Pax & K. Hoffm.	Euphorbiaceae
*Maranthes chrysophylla*	(Oliv.) Prance	Chrysobalanaceae
*Maranthes glabra*	(Oliv.) Prance	Chrysobalanaceae
*Mareyopsis longifolia*	(Pax) Pax & K. Hoffm.	Euphorbiaceae
*Margaritaria discoidea*	(Baill.) G.L. Webster	Phyllanthaceae
*Markhamia lutea*	(Benth.) K. Schum.	Bignoniaceae
*Massularia acuminata*	(G. Don) Bullock ex Hoyle	Rubiaceae
*Memecylon afzelii*	G. Don	Melastomataceae
*Microdesmis puberula*	Hook. f. ex Planch.	Pandaceae
*Microdesmis zenkeri*	Pax	Pandaceae
*Milicia excelsa*	(Welw.) C.C. Berg	Moraceae
*Millettia laurentii*	De Wild.	Fabaceae
*Millettia sanagana*	Harms	Fabaceae
*Mitragyna stipulosa*	(DC.) Kuntze	Rubiaceae
*Monodora myristica*	(Gaertn.) Dunal	Annonaceae
*Monodora tenuifolia*	Benth.	Annonaceae
*Musanga cecropioides*	R. Br. ex Tedlie	Urticaceae
*Myrianthus arboreus*	P. Beauv.	Urticaceae
*Nauclea diderrichii*	(De Wild.) Merr.	Rubiaceae
*Nauclea pobeguinii*	(Hua ex Pobég.) Merr.	Rubiaceae
*Nesogordonia papaverifera*	(A. Chev.) Capuron ex N. Hallé	Malvaceae
*Ochna calodendron*	Gilg & Mildbr.	Ochnaceae
*Odyendyea gabonensis*	(Pierre) Engl.	Simaroubaceae
*Omphalocarpum elatum*	Miers	Sapotaceae
*Omphalocarpum lecomteanum*	Pierre ex Engl.	Sapotaceae
*Oncoba echinata*	Oliv.	Salicaceae
*Oncoba gilgiana*	Sprague	Salicaceae
*Oncoba glauca*	(P. Beauv.) Planch.	Salicaceae
*Oncoba welwitschii*	Oliv.	Salicaceae
*Ongokea gore*	(Hua) Pierre	Olacaceae
*Ophiobotrys zenkeri*	Gilg	Salicaceae
*Oxyanthus speciosus*	DC.	Rubiaceae
*Pancovia pedicellaris*	Radlk. & Gilg	Sapindaceae
*Panda oleosa*	Pierre	Pandaceae
*Parinari excelsa*	Sabine	Chrysobalanaceae
*Parkia bicolor*	A. Chev.	Fabaceae
*Pauridiantha callicarpoides*	(Hiern) Bremek.	Rubiaceae
*Pauridiantha efferata*	N. Hallé	Rubiaceae
*Pauridiantha floribunda*	(K. Schum. & K. Krause) Bremek.	Rubiaceae
*Pausinystalia macroceras*	(K. Schum.) Pierre	Rubiaceae
*Pentaclethra macrophylla*	Benth.	Fabaceae
*Pentadesma butyracea*	Sabine	Clusiaceae
*Persea americana*	Mill.	Lauraceae
*Petersianthus macrocarpus*	(P. Beauv.) Liben	Lecythidaceae
*Phyllocosmus africanus*	(Hook. f.) Klotzsch	Ixonanthaceae
*Phyllocosmus sessiliflorus*	Oliv.	Ixonanthaceae
*Picralima nitida*	(Stapf) T. Durand & H. Durand	Apocynaceae
*Piptadeniastrum africanum*	(Hook. f.) Brenan	Fabaceae
*Plagiostyles africana*	(Müll. Arg.) Prain	Euphorbiaceae
*Pouteria altissima*	(A. Chev.) Baehni	Sapotaceae
*Pouteria pierrei*	(A. Chev.) E.O. Beal	Sapotaceae
*Pseudospondias microcarpa*	(A. Rich.) Engl.	Anacardiaceae
*Psidium guajava*	L.	Myrtaceae
*Pteleopsis hylodendron*	Mildbr.	Combretaceae
*Pterocarpus mildbraedii*	Harms	Fabaceae
*Pterocarpus soyauxii*	Taub.	Fabaceae
*Pterygota bequaertii*	De Wild.	Malvaceae
*Pycnanthus angolensis*	(Welw.) Warb.	Myristicaceae
*Quassia undulata*	(Guill. & Perr.) D. Dietr.	Simaroubaceae
*Raphia monbuttorum*	Drude	Arecaceae
*Raphia regalis*	Becc.	Arecaceae
*Rauvolfia caffra*	Sond.	Apocynaceae
*Rauvolfia vomitoria*	Wennberg	Apocynaceae
*Rhabdophyllum bracteolatum*	(Gilg) Farron	Ochnaceae
*Ricinodendron heudelotii*	(Baill.) Pierre ex Pax	Euphorbiaceae
*Rinorea longifolia*	De Wild.	Violaceae
*Rinorea oblongifolia*	(C.H. Wright) Marquand ex Chipp	Violaceae
*Rinorea spongiocarpa*	Achound.	Violaceae
*Rinorea subsessilis*	M. Brandt	Violaceae
*Rinorea welwitschii*	Kuntze	Violaceae
*Rothmannia lateriflora*	(K. Schum.) Keay	Rubiaceae
*Rothmannia lujae*	(De Wild.) Keay	Rubiaceae
*Santiria trimera*	(Oliv.) Aubrév.	Burseraceae
*Sapium ellipticum*	(Hochst.) Pax	Euphorbiaceae
*Schrebera arborea*	A. Chev.	Oleaceae
*Scottellia klaineana*	Pierre	Achariaceae
*Sorindeia grandifolia*	Engl.	Anacardiaceae
*Spathodea campanulata*	P. Beauv.	Bignoniaceae
*Staudtia kamerunensis*	Warb.	Myristicaceae
*Sterculia tragacantha*	Lindl.	Malvaceae
*Streblus usambarensis*	(Engl.) C.C. Berg	Moraceae
*Strephonema pseudo-cola*	A. Chev.	Combretaceae
*Strombosia grandifolia*	Hook. f.	Olacaceae
*Strombosia pustulata*	Oliv.	Olacaceae
*Strombosia scheffleri*	Engl.	Olacaceae
*Strombosia zenkeri*	Engl.	Olacaceae
*Strombosiopsis tetrandra*	Engl.	Olacaceae
*Symphonia globulifera*	L. f.	Clusiaceae
*Synsepalum brevipes*	(Baker) T.D. Penn.	Sapotaceae
*Synsepalum dulcificum*	(Schumach. & Thonn.) Daniell	Sapotaceae
*Synsepalum longecuneatum*	De Wild.	Sapotaceae
*Syzygium guineense*	(Willd.) DC.	Myrtaceae
*Syzygium rowlandii*	Sprague	Myrtaceae
*Tabernaemontana citrifolia*	L.	Apocynaceae
*Tabernaemontana crassa*	Benth.	Apocynaceae
*Tapura africana*	Oliv.	Dichapetalaceae
*Teclea afzelii*	Engl.	Rutaceae
*Terminalia superba*	Engl. & Diels	Combretaceae
*Tessmannia africana*	Harms	Fabaceae
*Tetracera alnifolia*	Willd.	Dilleniaceae
*Tetracera stuhlmanniana*	Gilg	Dilleniaceae
*Tetrapleura tetraptera*	(Schumach. & Thonn.) Taub.	Fabaceae
*Tetrorchidium didymostemon*	(Baill.) Pax & K. Hoffm.	Euphorbiaceae
*Treculia africana*	Decne. ex Trécul	Moraceae
*Trema orientalis*	(L.) Blume	Cannabaceae
*Tricalysia anomala*	E.A. Bruce	Rubiaceae
*Tricalysia crepiniana*	De Wild. & T. Durand	Rubiaceae
*Tricalysia discolor*	Brenan	Rubiaceae
*Trichilia monadelpha*	(Thonn.) J.J. de Wilde	Meliaceae
*Trichilia prieuriana*	A. Juss.	Meliaceae
*Trichilia rubescens*	Oliv.	Meliaceae
*Trichilia tessmannii*	Harms	Meliaceae
*Trichilia welwitschii*	C. DC.	Meliaceae
*Trichoscypha acuminata*	Engl.	Anacardiaceae
*Trichoscypha arborea*	A. Chev.	Anacardiaceae
*Tridesmostemon omphalocarpoides*	Engl.	Sapotaceae
*Trilepisium madagascariense*	DC.	Moraceae
*Triplochiton scleroxylon*	K. Schum.	Malvaceae
*Turraeanthus africana*	(Welw. ex C. DC.) Pellegr.	Meliaceae
*Uapaca acuminata*	(Hutch.) Pax & K. Hoffm.	Phyllanthaceae
*Uapaca guineensis*	Müll. Arg.	Phyllanthaceae
*Uapaca paludosa*	Aubrév. & Leandri	Phyllanthaceae
*Uapaca staudtii*	Pax	Phyllanthaceae
*Uapaca vanhouttei*	De Wild.	Phyllanthaceae
*Uvariopsis dioica*	(Diels) Robyns & Ghesq.	Annonaceae
*Vepris louisii*	G.C.C. Gilbert	Rutaceae
*Vitex grandifolia*	Gürke	Lamiaceae
*Vitex rivularis*	Gürke	Lamiaceae
*Vitex thyrsiflora*	Baker	Lamiaceae
*Vitex zenkeri*	Gürke	Lamiaceae
*Xylopia aethiopica*	(Dunal) A. Rich.	Annonaceae
*Xylopia hypolampra*	Mildbr.	Annonaceae
*Xylopia parviflora*	(Guill. & Perr.) Engl. & Diels	Annonaceae
*Xylopia quintasii*	Pierre ex Engl. & Diels	Annonaceae
*Xylopia rubescens*	Oliv.	Annonaceae
*Zanthoxylum gilletii*	(De Wild.) P.G. Waterman	Rutaceae
*Zanthoxylum heitzii*	(Aubrév. & Pellegr.) P.G.Waterman	Rutaceae

**Table 2.** Ten most species rich families in the Dja Faunal Reserve (excluding non identified taxa (sp.)).

Position	Family	Species richness
1	Fabaceae	37
2	Rubiaceae	24
3	Malvaceae	23
4	Euphorbiaceae	18
5	Sapotaceae	16
6	Annonaceae	15
7	Meliaceae	14
8	Phyllanthaceae	13
9	Putranjivaceae	11
10	Clusiaceae	9

**Table 3.** Ten most abundant families in terms of individuals inventoried.

Family	Family abundance
Fabaceae	1203
Phyllanthaceae	1011
Olacaceae	830
Annonaceae	826
Euphorbiaceae	794
Apocynaceae	775
Meliaceae	605
Rubiaceae	446
Burseraceae	436
Malvaceae	396

**Table 4.** Twenty most abundant species in the Dja Faunal Reserve.

Position	Species	Species abundance
1	*Tabernaemontana crassa*	633
2	*Petersianthus macrocarpus*	382
3	*Santiria trimera*	349
4	*Greenwayodendron suaveolens*	332
5	*Uapaca guineensis*	330
6	*Uapaca paludosa*	260
7	*Strombosiopsis tetrandra*	240
7	*Pentaclethra macrophylla*	240
7	*Plagiostyles africana*	240
8	*Desbordesia glaucescens*	210
9	*Trichilia rubescens*	206
10	*Dichostemma glaucescens*	197
11	*Rinorea oblongifolia*	189
12	*Anonidium mannii*	181
13	*Strombosia pustulata*	172
14	*Heisteria trillesiana*	156
15	*Coelocaryon preussii*	152
16	*Carapa procera*	136
17	*Anthonotha macrophylla*	134
18	*Corynanthe pachyceras*	131
19	*Trichoscypha acuminata*	128
20	*Antidesma laciniatum*	124

**Table 5.** Tree diversity and abundance among the nine transects in the Dja Faunal Reserve. Family and species are ordered alphabetically.

Family/species										
Plot number	L1	L2	L3	L4	L5	L6	L7	L8	L9	Total
** Achariaceae **		**2**	**2**	**1**		**2**		**1**		**8**
*Buchnerodendron speciosum*								1		1
*Lindackeria echinata*						1				1
*Scottellia klaineana*		2	2	1		1				6
** Agavaceae **	**2**	**1**	**1**			**1**				**5**
*Dracaena arborea*	2	1	1			1				5
** Anacardiaceae **	**61**	**60**	**43**	**42**	**26**	**32**	**32**	**22**	**21**	**339**
*Antrocaryon klaineanum*	1			1					1	3
*Antrocaryon micraster*									1	1
*Lannea welwitschii*		1		1	2	1	4	2	3	14
*Pseudospondias microcarpa*	14	3	6	3	2	6	3		2	39
*Sorindeia grandifolia*	19	29	16	13	5	4	7		3	96
*Sorindeia* sp.	6	4	6	4	4	6	1	11	4	46
*Trichoscypha acuminata*	20	22	15	17	13	15	11	8	7	128
*Trichoscypha arborea*	1	1		3			6	1		12
** Anisophylleaceae **	**1**			**2**	**2**	**2**		**3**		**10**
*Anopyxis klaineana*	1			2	2	2		3		10
** Annonaceae **	**136**	**112**	**103**	**69**	**75**	**97**	**64**	**99**	**71**	**826**
*Annickia affinis*	9	17	9	5	7	10	11	14	6	88
*Anonidium mannii*	53	22	19	3	21	14	9	13	27	181
*Cleistopholis glauca*		1		4	1	5				11
*Cleistopholis patens*	5	5	4	4	6	4	4	2	2	36
*Duguetia staudtii*	4	3	5	3	4	4	2	2	2	29
*Greenwayodendron suaveolens*	46	37	42	28	22	45	27	55	30	332
*Hexalobus crispiflorus*	6	3	1	1	1	1	1			14
*Monodora myristica*					1					1
*Monodora tenuifolia*	1	3		2	1	2				9
*Uvariopsis dioica*	1	3	2	8						14
*Xylopia aethiopica*	2	2	3		2	3	2			14
*Xylopia hypolampra*		1						1		2
*Xylopia parviflora*		4	2		3	2				11
*Xylopia quintasii*	6	6	12	7	5	2	7	9	3	57
*Xylopia rubescens*	1	4	4	2		5	1		1	18
*Xylopia* sp.	2	1		2	1			3		9
** Apocynaceae **	**73**	**145**	**36**	**79**	**133**	**124**	**71**	**82**	**32**	**775**
*Alstonia boonei*	5	5	1	4	10	7	5	6	2	45
*Funtumia africana*									1	1
*Funtumia elastica*	2	5	2	1	16	25	6	5	2	64
*Landolphia* sp.				3	2					5
*Picralima nitida*	2	2		4	5	2	1	1		17
*Rauvolfia caffra*	1							1		2
*Rauvolfia vomitoria*			1	1		2		1		5
*Strophanthus* sp.						1				1
*Tabernaemontana citrifolia*		1								1
*Tabernaemontana crassa*	63	132	32	66	100	87	59	67	27	633
*Tabernanthe* sp.								1		1
** Arecaceae **			**10**	**2**	**17**	**38**	**1**		**25**	**93**
*Raphia monbuttorum*			10	2	17	38	1			68
*Raphia regalis*									25	25
** Asteraceae **							**1**			**1**
*Vernonia* sp.							1			1
** Bignoniaceae **	**2**	**2**	**1**	**8**	**3**	**8**	**3**	**1**	**3**	**31**
*Fernandoa adolfi-friderici*	1	2	1	1		2		1		8
*Markhamia lutea*	1			7	3	6			2	19
*Spathodea campanulata*							3		1	4
** Boraginaceae **	**1**			**1**	**2**	**1**	**2**	**2**		**9**
*Cordia aurantiaca*				1	2	1				4
*Cordia platythyrsa*	1						2	2		5
** Burseraceae **	**64**	**63**	**50**	**43**	**46**	**51**	**30**	**40**	**49**	**436**
*Canarium schweinfurthii*	1	1			1	2	2	2		9
*Dacryodes buettneri*					10	1				11
*Dacryodes edulis*	17	6	2	4	10	14	2	1	4	60
*Dacryodes klaineana*				1						1
*Dacryodes* sp.	1		4		1					6
*Santiria trimera*	45	56	44	38	24	34	26	37	45	349
** Calophyllaceae **	**6**	**4**	**7**	**2**	**4**	**4**	**6**	**3**	**3**	**39**
*Mammea africana*	6	4	7	2	4	4	6	3	3	39
** Cannabaceae **	**69**	**28**	**13**	**10**	**11**	**35**	**24**	**9**	**16**	**215**
*Celtis adolfi-friderici*									1	1
*Celtis gomphophylla*			1							1
*Celtis tessmannii*	19	17	5	5	11	7	19	8	11	102
*Celtis zenkeri*	50	11	7	5		28	5	1	1	108
*Trema orientalis*									3	3
** Celastraceae **		**8**	**7**	**5**	**3**	**7**				**30**
*Salacia* sp.		8	7	5	3	7				30
** Centroplacaceae **	**6**	**6**	**12**	**4**		**1**			**5**	**34**
*Centroplacus glaucinus*	6	6	12	4		1			5	34
** Chrysobalanaceae **	**17**	**11**	**7**	**12**	**3**		**6**	**4**	**19**	**79**
*Acioa* sp.			1		1					2
*Maranthes chrysophylla*			1	8			2		14	25
*Maranthes glabra*	12	4	3	2			2	2	1	26
*Maranthes* sp.	5	4	2	2	1		1	1	2	18
*Parinari excelsa*		2			1		1	1	2	7
*Parinari* sp.		1								1
** Clusiaceae **	**22**	**20**	**38**	**16**	**8**	**5**	**19**	**19**	**15**	**162**
*Allanblackia floribunda*	13	5	6	8	6	1	8	2	10	59
*Allanblackia gabonensis*								1		1
*Garcinia gnetoides*			2					4		6
*Garcinia kola*	1									1
*Garcinia mannii*	4	12	9	8	2	2	5	2	1	45
*Garcinia punctata*									1	1
*Garcinia smeathmannii*			11							11
*Garcinia* sp.		2						1		3
*Pentadesma butyracea*							2	1		3
*Symphonia globulifera*	4	1	10			2	4	8	3	32
** Combretaceae **	**3**	**8**	**2**	**10**	**12**	**5**	**6**	**4**	**5**	**55**
*Combretum* sp.		5	1	2						8
*Pteleopsis hylodendron*	1		1	4	6		1	1	1	15
*Strephonema pseudo-cola*						1				1
*Strephonema* sp.		1				3				4
*Terminalia superba*	2	2		4	6	1	5	3	4	27
** Dichapetalaceae **		**1**								**1**
*Tapura africana*		1								1
** Dilleniaceae **		**2**		**5**		**3**				**10**
*Tetracera alnifolia*		1				3				4
*Tetracera stuhlmanniana*		1		5						6
** Ebenaceae **	**17**	**14**	**3**	**3**	**17**	**16**	**10**	**10**	**14**	**104**
*Diospyros bipindensis*	1						3		1	5
*Diospyros cinnabarina*					3					3
*Diospyros conocarpa*		1								1
*Diospyros crassiflora*				1			3	2	7	13
*Diospyros hoyleana*	4	4	2		5	5	1	6		27
*Diospyros* sp.	4	4	1	1	7	2	1	2	1	23
*Diospyros suaveolens*	8	5		1	2	9	2		5	32
** Euphorbiaceae **	**32**	**82**	**80**	**84**	**100**	**29**	**133**	**139**	**81**	**760**
*Cavacoa quintasii*				17						17
*Croton oligandrus*					1					1
*Crotonogyne preussii*						1				1
*Dichostemma glaucescens*	12	37	38	32	55	4	13		6	197
*Discoglypremna caloneura*	4		2	5	4	3	3		1	22
*Elaeophorbia grandifolia*					1					1
*Hymenocardia lyrata*	1			2	1				7	11
*Hymenocardia* sp.			1		1					2
*Klaineanthus gaboniae*		5	6	1	7	11	3	11	13	57
*Macaranga barteri*						2			3	5
*Macaranga occidentalis*		2		2						4
*Macaranga* sp.		4	2		2		1	1	2	12
*Macaranga spinosa*	11	10	2	1	12	4	12		1	53
*Maprounea membranacea*							3			3
*Maprounea* sp.									1	1
*Mareyopsis longifolia*		21	28	21	5		1	22	1	99
*Plagiostyles africana*	1	2		1	4		86	103	43	240
*Ricinodendron heudelotii*	3				1	4	2	2	3	15
*Sapium ellipticum*		1	1	1	1					4
*Tetrorchidium didymostemon*				1	5		9			15
** Fabaceae **	**181**	**156**	**164**	**123**	**130**	**107**	**157**	**84**	**101**	**1203**
*Afzelia bella*						1				1
*Afzelia bipindensis*					1			2	1	4
*Albizia adianthifolia*	3	2	1		6	2	3			17
*Albizia ferruginea*						1				1
*Albizia glaberrima*	1	3	1			1	3	4		13
*Albizia gummifera*							3		4	7
*Albizia* sp.	5	1			1				1	8
*Albizia zygia*		1	1				1		1	4
*Amphimas ferrugineus*	2									2
*Amphimas pterocarpoides*	1			1		1		2	1	6
*Angylocalyx pynaertii*	8	6	13	10	3	9	13	1	12	75
*Angylocalyx* sp.		1	1	1						3
*Anthonotha cladantha*	7	13	18			1				39
*Anthonotha fragrans*								3		3
*Anthonotha macrophylla*	10	26	38	19	10	12	7	10	2	134
*Anthonotha* sp.							1	1		2
*Balanites wilsoniana*		1								1
*Baphia* sp.									1	1
*Calpocalyx dinklagei*	8	8	13	20	10	10	3	12	5	89
*Cylicodiscus gabunensis*	3	5	1	1	5		1	2	2	20
*Detarium macrocarpum*	1	1	1	3	3		2		1	12
*Dialium bipindense*			1							1
*Dialium dinklagei*	4	2	1		1					8
*Dialium guineense*		1		1		1	6	3	3	15
*Dialium zenkeri*	3	3	15	16	8	8	6	1	2	62
*Distemonanthus benthamianus*	15	4	2	1	13	7				42
*Entada gigas*	2	5	1	3		5				16
*Erythrophleum suaveolens*	4	6	1	6	6	8	2	3		36
*Gilbertiodendron dewevrei*							52	1		53
*Gossweilerodendron joveri*		1								1
*Hylodendron gabunense*	19	12	8	5	9	8	9	3	4	77
*Hymenostegia afzelii*					1					1
*Millettia laurentii*			2							2
*Millettia sanagana*					2					2
*Millettia* sp.	1			2	1					4
*Newtonia* sp.							1			1
*Oddoniodendron* sp.									1	1
*Parkia bicolor*					3	1	1			5
*Pentaclethra macrophylla*	44	31	31	24	29	14	19	25	23	240
*Piptadeniastrum africanum*	7		1	1	1	2	9	2	7	30
*Pterocarpus mildbraedii*	16	12	1	3		4	2		3	41
*Pterocarpus soyauxii*	15	7	7	5	10	10	7	4	11	76
*Tessmannia africana*			4			1	6	5	15	31
*Tetrapleura tetraptera*	2	4	1	1	7				1	16
** Hypericaceae **							**3**			**3**
*Harungana madagascariensis*							3			3
** Irvingiaceae **	**34**	**40**	**49**	**62**	**42**	**43**	**34**	**44**	**37**	**385**
*Desbordesia glaucescens*	13	31	25	43	25	28	12	16	17	210
*Irvingia excelsa*			1						1	2
*Irvingia gabonensis*	11	4	15	8	10	4	12	10	8	82
*Irvingia grandifolia*	4	2	2	3	2	5	1	3	3	25
*Irvingia robur*							1			1
*Irvingia* sp.							1			1
*Klainedoxa gabonensis*	3	2	6	8	5	6	5	13	7	55
*Klainedoxa microphylla*	3	1					2	2	1	9
** Ixonanthaceae **			**4**					**1**	**3**	**8**
*Oubanguia* sp.			1							1
*Phyllocosmus africanus*								1	1	2
*Phyllocosmus sessiliflorus*			3							3
*Phyllocosmus* sp.									2	2
** Lauraceae **	**4**	**5**	**3**	**3**	**3**	**6**	**5**	**2**	**6**	**37**
*Beilschmiedia* sp.	4	5	3	3	2	6	1	2	6	32
*Persea americana*					1		4			5
** Lecythidaceae **	**101**	**47**	**19**	**8**	**81**	**51**	**34**	**20**	**21**	**382**
*Petersianthus macrocarpus*	101	47	19	8	81	51	34	20	21	382
** Lepidobotryaceae **	**15**	**21**	**12**	**5**	**4**	**5**	**7**	**13**	**2**	**84**
*Lepidobotrys staudtii*	15	21	12	5	4	5	7	13	2	84
** Loganiaceae **	**1**					**1**	**1**			**3**
*Strychnos* sp.	1					1	1			3
** Malvaceae **	**39**	**56**	**45**	**45**	**55**	**35**	**63**	**49**	**31**	**418**
*Bombax brevicuspe*						1	3	5		9
*Bombax buonopozense*	1	1					2	2		6
*Ceiba pentandra*	2			1	1		1		2	7
*Chlamydocola chlamydantha*	1		4	2	2	2	1		1	13
*Cola acuminata*		6	9		13	1	3	5		37
*Cola altissima*								1		1
*Cola ballayi*		3			3		4		6	16
*Cola flavo-velutina*				1						1
*Cola lateritia*	4	5	3	1	11	6	6	2		38
*Cola* sp.	8	4	10	16	1	1	7	12	2	61
*Cola verticillata*									1	1
*Desplatsia chrysochlamys*	4	12	4	7	9	6	6		1	49
*Desplatsia dewevrei*								3		3
*Duboscia macrocarpa*	6	11	11	10	4	9	11	6	8	76
*Eribroma oblongum*				2	1	2	3	1		9
*Glyphaea brevis*		1			5					6
*Grewia brunnea*	2									2
*Grewia coriacea*		2								2
*Grewia oligoneura*			1	2						3
*Grewia* sp.		2	1				1	4		8
*Leptonichia* sp.	1	1			1					3
*Nesogordonia papaverifera*							1		1	2
*Octolobus* sp.								1		1
*Pterygota bequaertii*	3	1						3		7
*Pterygota* sp.								1	1	2
*Sterculia tragacantha*	7	7	2	2	4	7	11	3	4	47
*Triplochiton scleroxylon*				1			3		4	8
** Melastomataceae **									**3**	**3**
*Memecylon afzelii*									3	3
** Meliaceae **	**97**	**98**	**57**	**23**	**56**	**65**	**71**	**67**	**71**	**605**
*Carapa procera*	36	27	19	1	16	22	5	7	3	136
*Entandrophragma angolense*	4			1	2	2				9
*Entandrophragma candollei*	4	2		1	4	3	5			19
*Entandrophragma cylindricum*	2	2	1	2		5		3	2	17
*Entandrophragma* sp.	4	2								6
*Guarea cedrata*	2	1			2	1	10	2	18	36
*Guarea thompsonii*	3	1		1	1		8		6	20
*Khaya ivorensis*		1								1
*Lovoa trichilioides*	7	10	6	2	1	4	4		2	36
*Trichilia monadelpha*		2		2			11	1	9	25
*Trichilia prieuriana*					2	1				3
*Trichilia rubescens*	33	46	24	8	26	26	25		18	206
*Trichilia* sp.	1	1	1	3	2	1	2	3	1	15
*Trichilia tessmannii*								49	1	50
*Trichilia welwitschii*		3					1			4
*Turraeanthus africana*	1		6	2				2	11	22
** Moraceae **	**75**	**17**	**7**	**9**	**10**	**3**	**21**	**4**	**30**	**176**
*Ficus exasperata*	2						7		2	11
*Ficus mucuso*							4	1	6	11
*Ficus sur*									1	1
*Milicia excelsa*	1		1	2	4		2		2	12
*Streblus usambarensis*			6	7					15	28
*Treculia africana*	7	5			4	2	2	3	2	25
*Trilepisium madagascariense*	65	12			2	1	6		2	88
** Myristicaceae **	**65**	**19**	**19**	**9**	**41**	**13**	**65**	**29**	**48**	**308**
*Coelocaryon botryoides*	1									1
*Coelocaryon preussii*	38	5	7	2	17	5	39	11	28	152
*Pycnanthus angolensis*	8	6	3	3	9	1	9	2	8	49
*Staudtia kamerunensis*	18	8	9	4	15	7	17	16	12	106
** Myrsinaceae **						**1**				**1**
*Embelia* sp.						1				1
** Myrtaceae **	**5**	**6**	**4**	**9**	**2**	**4**	**3**	**7**	**8**	**48**
*Eugenia* sp.	4	2	3	3		1		6	4	23
*Psidium guajava*							1			1
*Syzygium guineense*						1				1
*Syzygium rowlandii*		4	1	6	2	2	2	1	4	22
*Syzygium* sp.	1									1
** Ochnaceae **	**4**	**1**	**4**	**6**	**1**		**5**	**2**	**3**	**26**
*Ochna calodendron*							2			2
*Rhabdophyllum bracteolatum*	4	1	4	6	1		3	2	3	24
** Olacaceae **	**95**	**82**	**108**	**82**	**104**	**80**	**73**	**106**	**100**	**830**
*Heisteria trillesiana*	41	16	22	7	51	16	2		1	156
*Heisteria zimmereri*	2	4		1			26	24	35	92
*Ongokea gore*	5	1	4	2	4	1	2	2	1	22
*Strombosia grandifolia*	1	1	2	1		2	9	4	9	29
*Strombosia pustulata*	13	7	30	27	19	25	7	18	26	172
*Strombosia scheffleri*	4	16	21	9	1	14	5	12	1	83
*Strombosia* sp.		1				1				2
*Strombosia zenkeri*			5		27	2				34
*Strombosiopsis tetrandra*	29	36	24	35	2	19	22	46	27	240
** Oleaceae **				**1**						**1**
*Schrebera arborea*				1						1
** Pandaceae **	**9**	**10**	**6**	**12**	**6**	**13**	**8**	**8**	**14**	**86**
*Microdesmis puberula*				1						1
*Microdesmis* sp.	4	5	2		3	4	1		7	26
*Microdesmis zenkeri*			1							1
*Panda oleosa*	5	5	3	11	3	9	7	8	7	58
** Passifloraceae **		**4**			**4**	**1**	**9**	**1**		**19**
Barteria nigritana subsp. fistulosa		4			4	1	9	1		19
** Phyllanthaceae **	**127**	**170**	**90**	**77**	**124**	**68**	**107**	**171**	**77**	**1011**
*Antidesma laciniatum*	8	22	18	31	4	4	15	16	6	124
*Antidesma membranaceum*	3	5	2	6		2	9	4	4	35
*Antidesma* sp.		2		1				4		7
*Antidesma venosum*					2	1		11	1	15
*Bridelia grandis*				2		1	9	4		16
*Bridelia micrantha*	9	2	2		1	1	1			16
*Keayodendron bridelioides*	5	4	3	5	4	4	1	7	2	35
*Maesobotrya klaineana*	6	11	3	4	5	4	3	8	4	48
*Maesobotrya* sp.									3	3
*Margaritaria discoidea*	1	1	1		3		13		1	20
*Uapaca acuminata*	16	21	2		19	3		7		68
*Uapaca guineensis*	18	72	37	24	12	22	31	87	27	330
*Uapaca paludosa*	60	30	22	4	74	25	13	12	20	260
*Uapaca staudtii*						1	3	10	6	20
*Uapaca vanhouttei*	1						9	1	3	14
** Putranjivaceae **	**35**	**17**	**84**	**61**	**13**	**4**	**30**	**46**	**30**	**320**
*Drypetes aframensis*		1		7			2			10
*Drypetes africana*								1		1
*Drypetes afzelii*				1						1
*Drypetes capillipes*				5						5
*Drypetes chevalieri*	6	1	10						5	22
*Drypetes gossweileri*	2		2							4
*Drypetes klainei*								5		5
*Drypetes laciniata*			1	1		1	1		3	7
*Drypetes molunduana*									1	1
*Drypetes paxii*	1									1
*Drypetes* sp.	22	14	66	41	12	2	27	40	20	244
*Drypetes staudtii*	4	1	5	6	1	1			1	19
** Rhamnaceae **	**1**	**7**	**17**	**13**	**4**	**3**	**2**	**3**	**5**	**55**
*Lasiodiscus mannii*	1	4	16	13	2	3		3	4	46
*Maesopsis eminii*		3	1		2		2		1	9
** Rubiaceae **	**34**	**29**	**64**	**61**	**30**	**40**	**59**	**57**	**72**	**446**
*Aidia micrantha*							17	12	52	81
*Aoranthe cladantha*	3			4	3	4		1	1	16
*Aulacocalyx jasminiflora*			2	1						3
*Belonophora coriacea*								2		2
*Bertiera racemosa*		1								1
*Brenania brieyi*	3	1	2	2			2	3	1	14
*Canthium* sp.	2	4	1		4	1		2	2	16
*Corynanthe pachyceras*	6	8	32	23	7	5	21	26	3	131
*Cuviera cuvieroides*	1	2	6	7	1	9				26
*Cuviera* sp.					1					1
*Heinsia crinita*	1	2	2	4						9
*Leptactina involucrata*							1			1
*Massularia acuminata*		1		1			1		1	4
*Mitragyna stipulosa*	2		5	2	1		1		4	15
*Nauclea diderrichii*					2		2	1	1	6
*Nauclea pobeguinii*							1	1	1	3
*Oxyanthus speciosus*	2						1			3
*Pauridiantha callicarpoides*				1						1
*Pauridiantha efferata*		1	5	1				2	2	11
*Pauridiantha floribunda*	3	1		5	1	1				11
*Pausinystalia macroceras*							1			1
*Pavetta* sp.	1									1
*Rothmannia lateriflora*	5	3	3		2	6	1	2		22
*Rothmannia lujae*	4	5	3	2	3	6	7	5	3	38
*Tricalysia anomala*				1		3	2			6
*Tricalysia crepiniana*				1	1					2
*Tricalysia discolor*	1		3	6	3	5	1		1	20
*Tricalysia* sp.					1					1
** Rutaceae **	**7**	**4**	**4**	**6**	**6**	**6**	**10**	**2**	**3**	**48**
*Clausena anisata*				1						1
*Teclea afzelii*						1	1			2
*Vepris louisii*				2			2			4
*Zanthoxylum gilletii*	7	3	4	3	6	5	3	2	3	36
*Zanthoxylum heitzii*		1					4			5
** Salicaceae **	**23**	**22**	**19**	**22**	**21**	**19**	**4**	**5**	**8**	**143**
*Casearia barteri*	11	6	16	2	4	2				41
*Homalium discophylum*	4	7		9	4	4	3	4		35
*Homalium le-testui*							1			1
*Homalium* sp.			2	1	1				2	6
*Oncoba echinata*	3	1								4
*Oncoba gilgiana*									1	1
*Oncoba glauca*	2	6	1	8	12	12		1	5	47
*Oncoba* sp.	1			2						3
*Oncoba welwitschii*						1				1
*Ophiobotrys* sp.	2									2
*Ophiobotrys zenkeri*		2								2
** Sapindaceae **	**33**	**34**	**23**	**67**	**14**	**35**	**34**	**21**	**9**	**270**
*Allophylus africanus*							4			4
*Blighia sapida*	18	13	8	8	6	19	2	5	2	81
*Blighia* sp.								1		1
*Blighia welwitschii*	3	11		12		1	9	6	5	47
*Chytranthus atroviolaceus*				1						1
*Chytranthus macrobotrys*									1	1
*Chytranthus* sp.	6	1	1	8	3	5	7	1		32
*Eriocoelum macrocarpum*	4	8	8	2		1		1		24
*Lecaniodiscus cupanioides*	2	1		5		2	9	7	1	27
*Pancovia pedicellaris*			6	31	5	7	3			52
** Sapotaceae **	**26**	**17**	**43**	**22**	**7**	**12**	**16**	**5**	**17**	**165**
*Afrosersalisia* sp.	3		1							4
*Autranella congolensis*			1							1
*Baillonella toxisperma*	3	2	1			1	1			8
*Chrysophyllum africanum*				1	2		3	1	3	10
*Chrysophyllum boukokoense*	1	1	1		1		1			5
*Chrysophyllum lacourtianum*	9	7	1	2	2	9	3	1		34
*Chrysophyllum pruniforme*	1		2		1	1				5
*Chrysophyllum* sp.									1	1
*Letestua* sp.	1									1
*Manilkara letouzei*	3	1	5	7			4	1		21
*Manilkara obovata*			2							2
*Manilkara* sp.		1		1						2
*Omphalocarpum elatum*	1						1			2
*Omphalocarpum lecomteanum*				2						2
*Pouteria altissima*		1		1		1		1	2	6
*Pouteria pierrei*								1		1
*Pouteria* sp.									1	1
*Synsepalum brevipes*				1					3	4
*Synsepalum dulcificum*			23							23
*Synsepalum longecuneatum*	2	3	3	3			2		1	14
*Tridesmostemon omphalocarpoides*	2	1	3	4	1		1		6	18
** Simaroubaceae **	**8**	**2**	**1**	**1**	**2**	**3**		**6**	**5**	**28**
*Odyendyea gabonensis*	8	2	1	1	2	2		6	5	27
*Quassia undulata*						1				1
** Urticaceae **	**17**	**14**	**1**	**5**	**12**	**6**	**45**	**2**	**59**	**161**
*Musanga cecropioides*	12			2	7	1	21		57	100
*Myrianthus arboreus*	5	14	1	3	5	5	24	2	2	61
** Verbenaceae **	**3**	**5**	**1**	**4**		**1**	**6**	**2**	**3**	**25**
*Vitex grandifolia*						1				1
*Vitex rivularis*	2	5		3			1			11
*Vitex* sp.	1		1					2	2	6
*Vitex thyrsiflora*				1						1
*Vitex zenkeri*							5		1	6
** Violaceae **	**26**	**16**	**18**	**13**	**3**	**11**	**30**	**50**	**28**	**195**
*Rinorea longifolia*	1									1
*Rinorea oblongifolia*	23	15	18	12	3	11	30	49	28	189
*Rinorea spongiocarpa*		1								1
*Rinorea subsessilis*				1				1		2
*Rinorea welwitschii*	2									2
** Vochysiaceae **	**2**	**4**	**18**		**1**	**3**	**1**		**2**	**31**
*Erismadelphus exsul*	2	4	18		1	3	1		2	31
**Total**	**1579**	**1472**	**1299**	**1147**	**1238**	**1100**	**1311**	**1245**	**1155**	**11546**
